# The effect of visceral obesity on clinicopathological features in patients with endometrial cancer: a retrospective analysis of 200 Chinese patients

**DOI:** 10.1186/s12885-016-2230-4

**Published:** 2016-03-11

**Authors:** Shuang Ye, Hao Wen, Zhaoxia Jiang, Xiaohua Wu

**Affiliations:** Department of Gynecologic Oncology, Fudan University Shanghai Cancer Center, Shanghai, China; Department of Oncology, Shanghai Medical College, Fudan University, No 270 Dong-an Road, Xuhui District, 200032 Shanghai, China; Department of Radiology, Fudan University Shanghai Cancer Center, Shanghai, China

**Keywords:** Endometrial cancer, Visceral adiposity, Body mass index, Clinicopathological features, Lymph node metastasis

## Abstract

**Background:**

To assess the effect of visceral adiposity on clinical and pathological characteristics in patients with endometrial cancer.

**Methods:**

A retrospective review of medical documentation was performed in surgically treated endometrial cancer patients from January to November 2015 in our institution. The visceral adipose tissue (VAT) and subcutaneous adipose tissue (SAT) were measured at the level of umbilicus on single-slice computerized tomography. Visceral adiposity (VAT%) was calculated as VAT/(VAT + SAT).

**Results:**

A total of 200 cases were included in the study. Median age at diagnosis was 54 years old. Most patients presented with early-stage tumor (86.0 % for I + II) and endometrioid histology (90.5 %). Positive lymph node occurred in 11.0 % (22/200) of the patients with the median number of retrieved nodes as 25 (range, 4–56). The entire population had a median body mass index (BMI) of 24.7 kg/m^2^ and median VAT% of 31.89 %. BMI correlated with total adipose tissue (correlation coefficient = 0.667, *P* < 0.001), but not with VAT% (*P* = 0.495). Viscerally obese patients tended to be old and post-menopausal (*P* < 0.001; *P* = 0.003). Nodal metastasis and extrauterine disease were more commonly reported in patients with high VAT% (6.0 % vs. 16.0 %, *P* = 0.024; 9.0 % vs. 19.0 %, *P* = 0.042, respectively). Univariate and multivariate logistic regressions were performed to discern the contribution of variable factors on the lymph node metastasis. Grade (HR = 15.41, 95 % CI = 1.60–148.76; *P* = 0.018), lympho-vascular invasion (HR = 449.61, 95 % CI = 31.27–6463.93; *P* < 0.001) and high VAT% (HR = 6.37, 95 % CI = 1.42–28.69; *P* = 0.016) retained statistical significance for predicting lymph node metastasis.

**Conclusions:**

Viscerally obese patients were more likely to be old and have positive lymph node as well as extrauterine disease. Grade, lympho-vascular invasion presence and visceral adiposity were predictors of nodal disease.

## Background

Endometrial cancer is the most common gynecologic malignancy in the United States [1]. Although less common in China, endometrial cancer has been in upward tendency [2], in parallel with the average body weight [3]. Obesity is a well-established risk factor for endometrial carcinoma [4, 5]. Recently, several investigators have explored the impact of obesity on prognostic features of endometrial cancer, primarily using measurements of body weight and indices of relative weight as an indicator of overall adiposity [6–12].

Body mass index (BMI) is commonly used in the definition and criteria of obesity. However, it is an imperfect measurement of body fat distribution that fails to distinguish between fat and muscle, and between visceral and subcutaneous fat [13]. Subcutaneous adipose tissue (SAT) and visceral adipose tissue (VAT) are different in cellular, molecular, physiological, clinical and prognostic perspectives [14]. Measurement of VAT has become an important consideration and has shown to be one of the most metabolically active fat compartments [14]. Given that most above-mentioned studies utilized BMI as measure of obesity and results were conflicting, we felt it necessary to investigate how VAT correlate with clinicopathological features of endometrial cancer.

Our institution is located in Shanghai, where the incidence of endometrial cancer increased with overall annual percent changes of 1.66 during the past 30 years [15]. We conducted this single-institutional retrospective study mainly for two purposes: firstly, to evaluate the correlation between BMI and SAT/VAT; secondly, to assess the role of adiposity in clinical and histopathologic outcomes of endometrial cancer.

## Methods

### Study patients and data collection

**Fig. 1:**
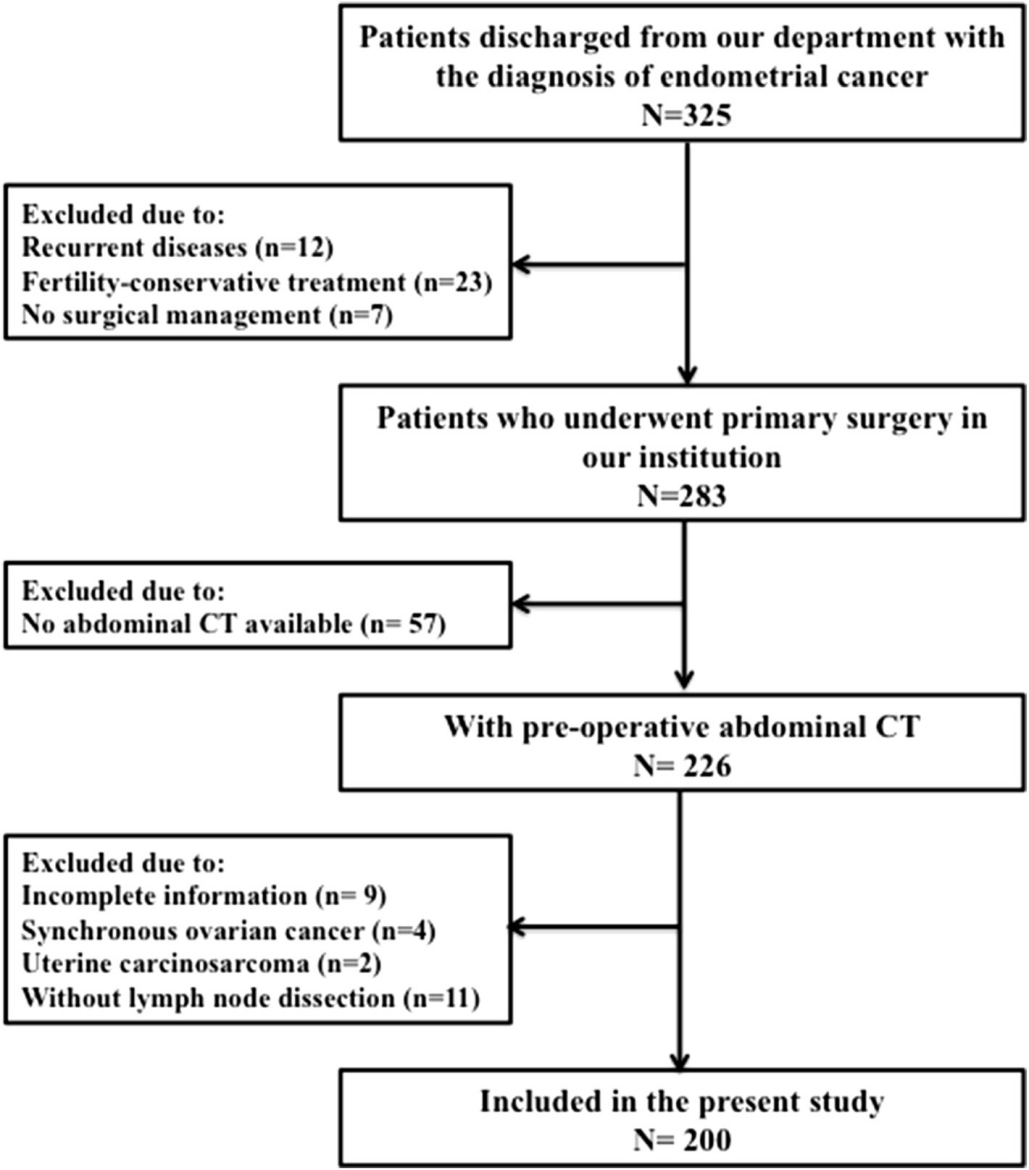
Schematic of patients included in the present study. On searching the electronic medical record database, 325 endometrial cancer patients were discharged from our department from 1 January to 1 November 2015. A total of 283 patients underwent primary surgery during the study period. Among them, 83 cases were excluded due to the following reasons: no available CT scan (*n* = 57), incomplete information (*n* = 9), concurrent primary ovarian cancer (*n* = 4), uterine carcinosarcoma (*n* = 2) and lymphadenectomy not performed (*n* = 11)

This study was approved by the ethics committee of Fudan University Shanghai Cancer Center. We searched the electronic medical record database to identify all the patients discharged from our department with the chief diagnosis of endometrial cancer from January 2015 to November 2015. Patients eligible for study inclusion fulfilled the following criteria: [1] patients underwent primary surgery treatment; [2] diagnosis of endometrial cancer confirmed by pathology; [3] pre-operative abdominal Computerized Tomography (CT) images available. Figure 1 presents the flow chart of patients throughout the study. A total of 200 patients were identified for further analyses. All the included patients gave their written informed consent.

A comprehensive review of medical documentation was then performed by a well-trained gynecologic oncologist. Data collection included age at diagnosis, menopausal state, comorbid conditions, BMI (calculated as weight (kg)/[height (m)]^2^), peritoneal cytology, tumor size (large tumor diameter recorded in the pathology report), histologic subtype, grade, myometrial invasion depth, presence of extrauterine disease, lymph node status, number of retrieved and positive lymph nodes, and International Federation of Gynecology and Obstetrics (FIGO) stage. In our institution, endometrial cancer patients usually receive complete staging surgery, including peritoneal cytology, total abdominal hysterectomy, bilateral salpingo-oophorectomy, and pelvic and para-aortic lymphadenectomy. All the patients were staged by the FIGO 2014 staging system [16].

In our routine practice, one surgical specimen is usually reviewed by two pathologists. Diagnosis was mainly dependent on the original pathology reports and pathology review was not conducted in this study. Histological grade was described by a three-tier system: grade 1 (well differentiated), grade 2 (moderately differentiated), and grade 3 (poorly differentiated and undifferentiated). Serous carcinoma and clear cell carcinoma were not graded, but all considered as grade 3.

### Adiposity measurement

**Fig. 2:**
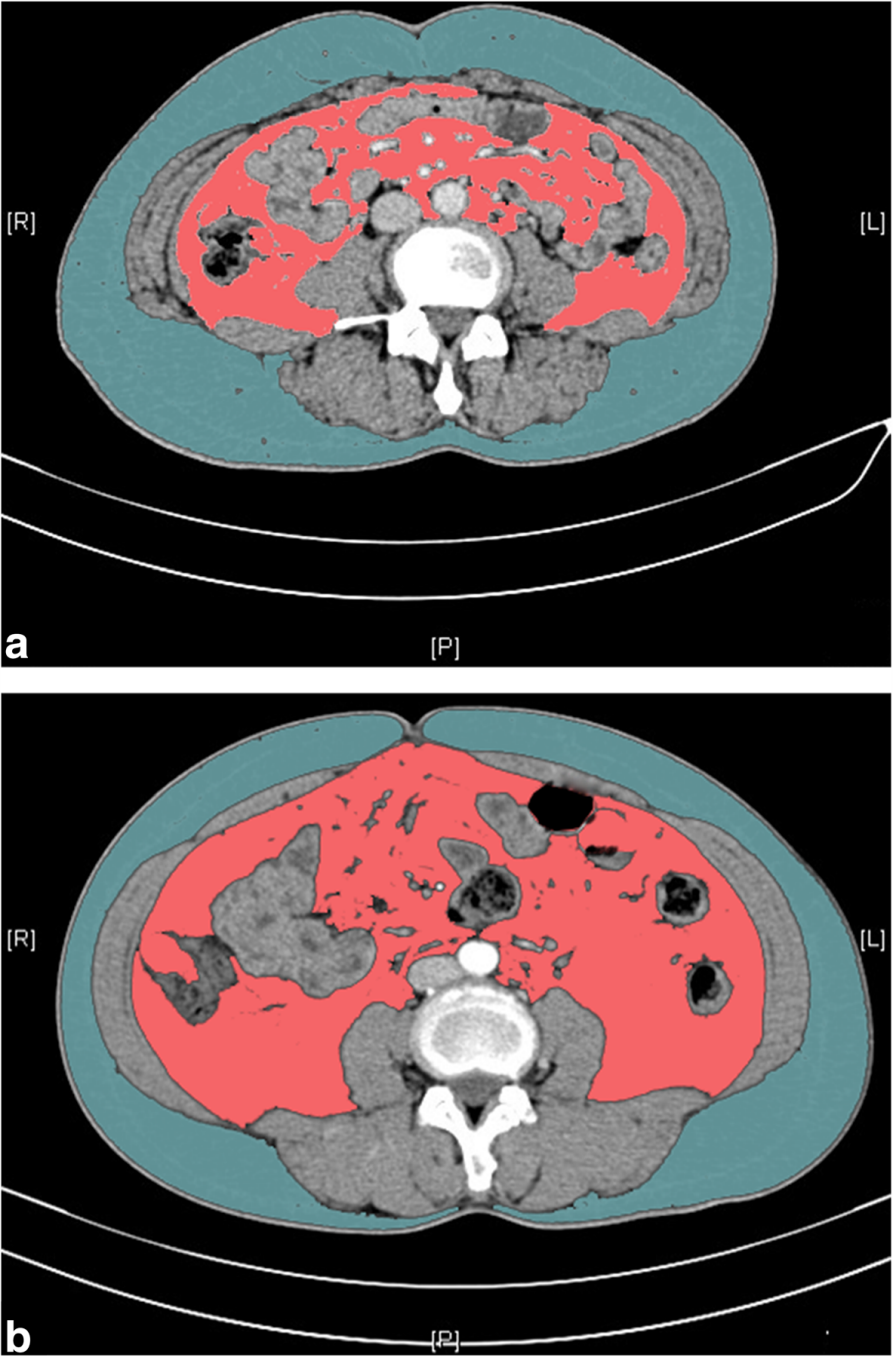
Measurements of visceral (Pink color) and subcutaneous (blue color) adipose tissue on computerized tomography images. **a**/**b** represents different body fat distribution: both patients’ body mass index is 24.7 kg/m^2^, while the visceral adipose tissue percentage (VAT%) is 30.3 % (**a**) and 56.95 % (**b**), respectively

Standard CT images based quantitative radiological measures have been regarded as the gold standard method for evaluating visceral adiposity [17]. As clearly shown in Fig. 2, VAT and SAT were measured at the level of umbilicus (approximately the level of L4-L5) [17]. SAT is defined as the fat area superficial to the abdominal muscular wall; VAT is deep to the muscular wall, consisting of the mesenteric, subperitoneal and retroperitoneal component. Total adipose tissue was obtained by adding SAT and VAT. The percentage of visceral fat to total fat area (VAT% = VAT/[VAT + SAT] × 100) was calculated to provide a single measure of abdominal fat [18]. ImageJ software [19] is employed for automatic calculation of adipose tissue area on the basis of pre-defined Hounsfield unit thresholds (-190 to -30). A single radiologist blinded to the clinicopathological characteristics was responsible for the measurement.

### Statistical analyses

Statistical Package for Social Science (SPSS) statistical software (Version 17.0, SPSS, Inc., Chicago, IL, USA) and GraphPad Prism (Version 5.0, GraphPad Software, Inc., La Jolla, CA, USA) were used for all the analyses. Continuous data were presented as median (range) and categorical data as proportions. Parametric Student *t* tests were employed in evaluating continuous variables while Chi-squared tests for categorical counterpart. Locally weighted scatter plot smoothing curves and Pearson’s correlation analysis were applied in comparison between obesity parameters (BMI, total adipose tissue, SAT, VAT and VAT%). The associations between different variables were assessed using univariate and multivariate logistic regression analysis, and hazard ratio (HR) with 95 % confidence interval (CI) was calculated. All of the *P* values reported were two-sided, and a value of *P* < 0.05 was considered statistically significant unless specified.

## Results

### Patient characteristics

Of 325 patients admitted into our hospital, 283 patients underwent primary surgery treatment (Fig. 1). Medical records were reviewed for the 283 consecutive patients. Among them, 57 patients didn't have available CT images due to outside hospital CT scan or other imaging modalities in our institution. We further excluded 26 cases: incomplete information (*n* = 9), synchronous primary cancers of the endometrium and ovary (*n* = 4), uterine carcinosarcoma (*n* = 2) and no lymphadenectomy performed (*n* = 11). Consequently, our study population consisted of 200 patients.

Table 1 presents the clinical and pathological characteristics of the entire cohort. Median age at diagnose was 54 years old (range, 28–84) and premenopausal patients accounted for 41.5 % (83/200). Medical comorbidities existed in 28.5 % (57/200) and 13.5 % (27/200) patients for hypertension and diabetes, respectively. Most tumors were endometrioid histology (90.5 %) and well to moderately differentiated (85.5 %). Deep myometrial invasion was reported in 23.0 % (46/200) of the cases. Comprehensive nodal status evaluation revealed that 11.0 % (22/200) of the patients had lymph node metastasis with median number of retrieved nodes as 25 (range, 4–56). Patients tended to have early-stage disease: 78.5 % stage I, 7.5 % stage II, and 14.0 % stage III. The rate of positive peritoneal cytology and lympho-vascular invasion was 9.4 % (16/171) and 19.9 % (37/186), respectively.

**Table 1:** Clinicopathological features of the study cohort

Parameters	Cohort	VAT% < 31.89 %	VAT% ≥ 31.89 %	*P* value
	*N* = 200	*N* = 100	*N* = 100	
Median age (range), years	54 (28–84)	52 (28–82)	57 (34–84)	**<0.001** ^**a**^
Premenopausal patients (%)	83 (41.5 %)	52 (52.0 %)	31 (31.0 %)	**0.003** ^**b**^
Hypertension (%)	57 (28.5 %)	19 (19.0 %)	38 (38.0 %)	**0.003** ^**b**^
Diabetes (%)	27 (13.5 %)	9 (9.0 %)	18 (18.0 %)	0.063^b^
Positive peritoneal cytology (%)	16 (9.4 %) *n* = 171	10 (11.6 %) *n* = 86	6 (7.1 %) *n* = 85	0.305^b^
Histology				
Endometrioid (%)	181 (90.5 %)	92 (92.0 %)	89 (89.0 %)	0.381^c^
Serous (%)	13 (6.5 %)	4 (4.0 %)	9 (9.0 %)	
Clear cell (%)	4 (2.0 %)	3 (3.0 %)	1 (1.0 %)	
Mucinous (%)	2 (1.0 %)	1 (1.0 %)	1 (1.0 %)	
Histological grade				
Grade 1	86 (43.0 %)	48 (48.0 %)	38 (38.0 %)	0.069^b^
Grade 2	85 (42.5 %)	43 (43.0 %)	42 (42.0 %)	
Grade 3	29 (14.5 %)	9 (9.0 %)	20 (20.0 %)	
Deep myometrial invasion (≥50 %) (%)	46 (23.0 %)	18 (18.0 %)	28 (28.0 %)	0.093^b^
Average tumor size (range), cm	3.14 (0.2–20)	3.19 (0.3–12)	3.09 (0.2–20)	0.791^a^
	*n* = 165^d^	*n* = 84^d^	*n* = 81^d^	
LN metastasis	22 (11.0 %)	6 (6.0 %)	16 (16.0 %)	**0.024** ^**b**^
Median No. retrieved LNs (range)	25 (4–56)	26 (4–55)	24 (4–56)	0.160^a^
Lympho-vascular invasion (%)	37 (19.9 %) *n* = 186	17 (18.3 %) *n* = 93	20 (21.5 %) *n* = 93	0.582^b^
Extrauterine disease (%)	28 (14.0 %)	9 (9.0 %)	19 (19.0 %)	**0.042** ^**b**^
FIGO stage				
IA (%)	134 (67.0 %)	73 (73.0 %)	61 (61.0 %)	
IB (%)	23 (11.5 %)	11 (11.0 %)	12 (12.0 %)	
II (%)	15 (7.5 %)	7 (7.0 %)	8 (8.0 %)	
IIIA (%)	3 (1.5 %)	2 (2.0 %)	1 (1.0 %)	
IIIB (%)	2 (1.0 %)	1 (1.0 %)	1 (1.0 %)	
IIIC (%)	23 (11.5 %)	6 (6.0 %)	17 (17.0 %)	
Median BMI (range), kg/m^2^	24.7 (17.5–43.7)	24.6 (18.03–43.7)	24.83 (17.53–35.16)	0.280^a^
Median VAT (range), mm^2^	17338 (5990–37118)	13780 (5990–31708)	20187(6649–37188)	**<0.001** ^**a**^
Median SAT (range), mm^2^	35607 (10458–106902)	41395 (20347–106902)	30360 (10458–61074)	**<0.001** ^**a**^
Median VAT% (range)	31.89 (13.97–59.57)	25.99 (13.97–31.88)	38.78(31.90–59.57)	**<0.001** ^**a**^

^a^Student’s T test

^b^Pearson Chi-square test

^c^Likelihood ratio

^d^Not all patients have tumor size recorded in the pathology report. Tumor confined within endometrium was reported without tumor size in 14 cases (7 and 7 in high and low VAT% group, respectively). A total of 21 patients (9 and 12 in high and low VAT% group, respectively) did not have tumor lesion in the final pathology after dilation and curettage

*Abbreviations: LN* lymph node, *BMI* body mass index, *SAT* subcutaneous Adipose Tissue, *VAT* visceral adipose tissue, *VAT %* percentage of visceral adipose tissue

Bold value denotes P with statistical significance

### Associations between obesity-related indices

Measurements of obesity are listed in the bottom part of Table 1. The median BMI for the entire cohort was 24.7 kg/m^2^, falling into the overweight category (BMI, 23.0–24.9 kg/m^2^) per World Health Organization guideline for Asia-Pacific populations [20]. VAT accounted for 31.89 % of the total adipose tissue, ranging from 13.97 % to 59.57 %.

**Fig. 3:**
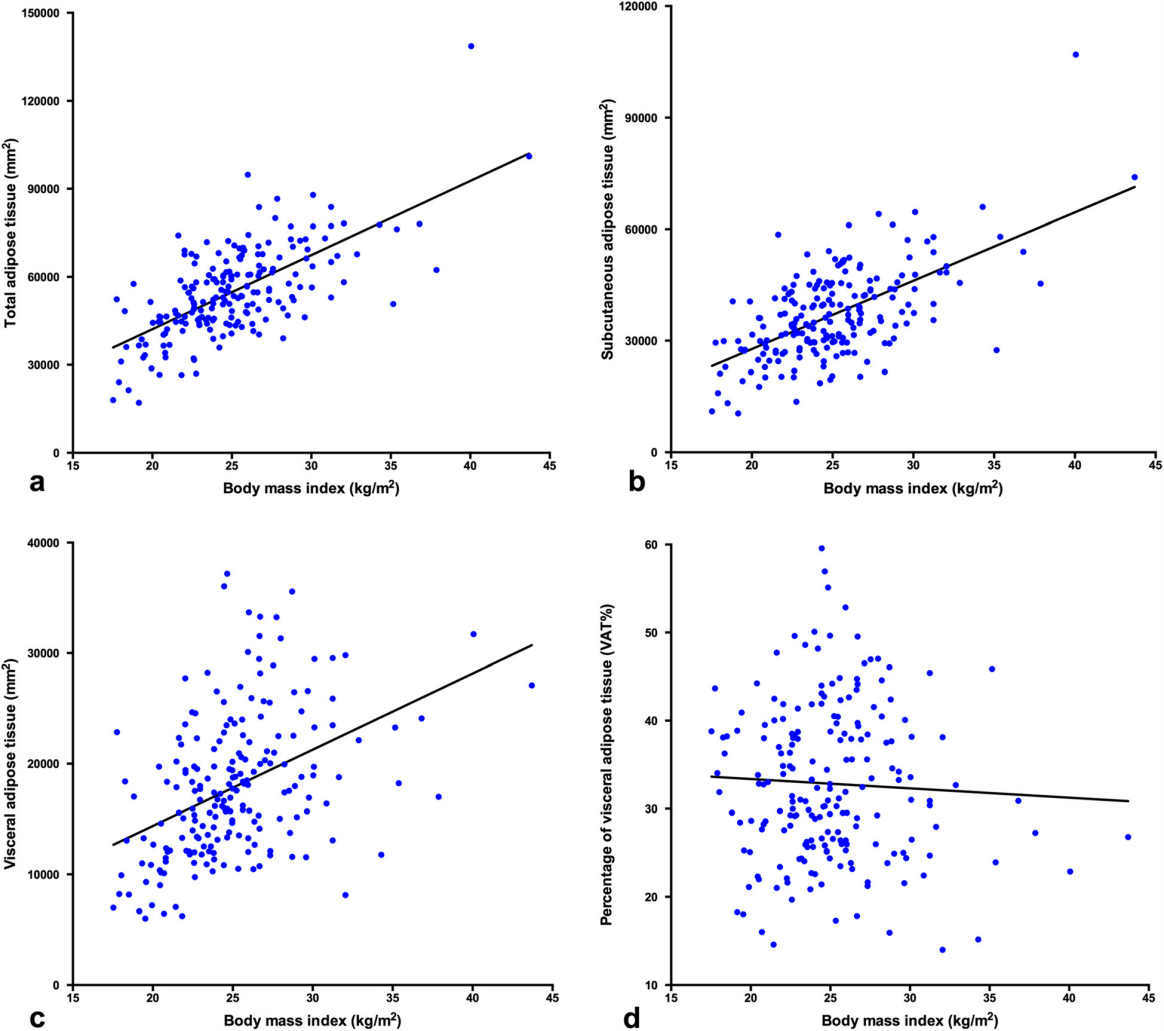
Scatter-plots of body mass index versus different fat distribution parameters. Total adipose tissue (**a**), subcutaneous adipose tissue (**b**), visceral adipose tissue (**c**), and visceral adipose tissue percentage (**d**)

We looked into the associations between obesity-related variables, which is presented in Table 2. BMI correlated well with total adipose tissue (correlation coefficient = 0.667) with statistical significance (*P* < 0.001). Not surprisingly, no correlation was found between BMI and VAT% (*P* = 0.495). Figure 3 illustrates scatter plots of BMI versus total adipose tissue, SAT, VAT and VAT% with locally weighted smoothing curves fitted in the plots.

**Table 2:** Correlations between obesity-related variables

		BMI	Total	SAT	VAT	VAT%
BMI	Correlation coefficient					
	*P* value					
Total	Correlation coefficient	0.667^a^				
	*P* value	<0.001				
SAT	Correlation coefficient	0.610^a^	0.913^a^			
	*P* value	<0.001	<0.001			
VAT	Correlation coefficient	0.428^a^	0.646^a^	0.279^a^		
	*P* value	<0.001	<0.001	<0.001		
VAT%	Correlation coefficient	−0.049	−0.123	−0.504^a^	0.654^a^	
	*P* value	0.495	0.082	<0.001	<0.001	

^a^Correlation is significant at the 0.01 level (2-tailed)

*Abbreviations: BMI* body mass index, *Total* total adipose tissue, *SAT* subcutaneous adipose tissue, *VAT* visceral adipose tissue, *VAT%* percentage of visceral adipose tissue

### Association between VTA and parameters

The entire cohort patients were dichotomized into two groups based on the median value of VAT% (results shown in Table 1). Viscerally obese patients were more likely to be old and post-menopausal (*P* < 0.001, *P* = 0.003, respectively). Hypertension was more commonly reported in the patients with high VAT% (*P* = 0.003). Regarding pathological features, correlations were noted in lymph node metastasis (6.0 % vs. 16.0% for low and high VAT% patients; *P* = 0.024) and extrauterine disease (9.0 % vs. 19.0 % for low and high VAT%; *P* = 0.042), respectively. VAT percentage was not statistically associated with peritoneal cytology, histology, grade, myometrial invasion depth, tumor size, lympho-vascular invasion and FIGO stage. It is worth mentioning that the numbers of retrieved lymph nodes were not significantly different between two groups (median number 26 vs. 24; *P* = 0.160).

Given the controversial role of lymphadenectomy in endometrial cancer, we further performed univariate and multivariate logistic regression to discern the contribution of variable factors on the lymph node metastasis (Tables 3 and 4). Given that tumor less than 2 cm has been suggested as indicative of low-risk for nodal metastases, tumor was dichotomized at the level of 2cm [21, 22]. When nodal disease was the dependent variable in univariate analysis, the following parameters were statistically significant: grade 3 (*P* = 0.001), tumor ≥2 cm (*P* = 0.010), deep myometrial invasion (*P* < 0.001), lympho-vascular invasion presence (*P* < 0.001), and high VAT% (*P* = 0.029). On multivariate analysis, lymph node metastasis was significantly associated with grade (HR = 15.41, 95 % CI = 1.60–148.76; *P* = 0.018), lympho-vascular invasion (HR = 449.61, 95 % CI = 31.27–6463.93; *P* < 0.001) and high VAT% (HR = 6.37, 95 % CI = 1.42–28.69; *P* = 0.016).

**Table 3:** Univariate analysis of factors predictive of lymph node metastasis

Univariate				
Parameters	Category	Cases	HR (95 % CI)	*P* value
Age	<54	102	1.58 (0.64–3.89)	0.319
	≥54	98		
BMI	<24.7	101	1.02 (0.42–2.48)	0.960
	≥24.7	99		
Cytology	Negative	155	1.44 (0.30–6.99)	0.652
	Positive	16		
Grade	1 + 2	171	5.47 (2.08–14.41)	**0.001**
	3	29		
Tumor size	<2cm	69	14.59 (1.92–111.14)	**0.010**
	≥2cm	117		
Histology	Endometrioid	181	1.95 (0.90–4.20)	0.090
	Non-endometrioid	19		
Myometrial invasion depth	<50 %	154	10.16 (3.82–26.99)	**<0.001**
	≥50 %	46		
LVSI	Negative	149	86.47 (18.58–402.47)	**<0.001**
	Positive	37		
Extrauterine disease	No	172	Not applicable^a^	0.994
	Yes	28		
VAT%	<31.89	100	2.98 (1.12–7.98)	**0.029**
	≥31.89	100		

^a^Estimation terminated at iteration number 20 because maximum iteration has been reached. Final solution cannot be found

*Abbreviations: HR* hazard ratio, *CI* confidence interval, *BMI* body mass index, *LVSI* lympho-vascular invasion, *VAT%* percentage of visceral adipose tissue

Bold value denotes P with statistical significance

**Table 4:** Analysis of factors predictive of lymph node metastasis

Multivariate		
Parameters	HR (95 % CI)	*P* value
Grade	15.41 (1.60–148.76)	**0.018**
Tumor size	0.22 (0.04–1.42)	0.112
Myometrial invasion depth	1.40 (0.28–6.95)	0.679
LVSI	449.61 (31.27–6463.93)	**<0.001**
VAT%	6.37 (1.42–28.69)	**0.016**

*Abbreviations: HR* hazard ratio, *CI* confidence interval, *LVSI* lympho-vascular invasion, *VAT%* percentage of visceral adipose tissue

Bold value denotes P with statistical significance

## Discussion

The effect of obesity on endometrial cancer has been of interest for decades. Several publications focused on the impact of obesity on clinical and pathological features of endometrial cancer, yet leading to inconsistent findings [6, 7, 9–12]. Everett et al [6] found that patients with a BMI of >40 kg/m^2^ frequently had favorable stage I endometrial cancers. A population-based study from Norway investigated the relationship between BMI and a large panel of clinicopathological data [10]. In their series, high BMI was significantly associated with markers of non-aggressive disease, including low FIGO stage and low/intermediate grade [10]. In contrast, the Women’s Health Initiative study reported that neither BMI nor waist-to-hip ratio correlated with stage or grade of disease [11]. Meanwhile, the authors suggested that additional measurement of adiposity should be considered [11], which was one of the inspiration sources for the present study.

In this cohort of 200 patients with endometrial cancer, we found that patients with high VAT% were more apt to be old and post-menopausal with statistical significance. The differences in clinicopathological features between the patients with low and high VAT% lied in the incidence of positive nodes (6.0 % vs. 16.0 %; *P* = 0.024) and extrauterine disease (9.0 % vs. 19.0 %, *P* = 0.042). In addition, grade 3, lympho-vascular invasion presence and high VAT% and were independent predictive factors for lymph node metastasis after adjusting for other variables. We suggested that high VAT% might be a marker of aggressive disease, which however warrants further investigation and validation. Given the consideration and controversy of omitting lymphadenectomy in endometrial cancer, we hoped to collect more cases in our future study in order to establish a model, incorporating several prognostic variables (i.e. grade, lympho-vascular invasion and VAT%), to evaluate the risk of lymph node metastasis on individual basis.

Despite that no direct mechanism has ever been elucidated, recent works have improved our understanding of the association between visceral adiposity and carcinogenesis [23–25]. Compared to subcutaneous fat depots, visceral counterpart is considered to be more pro-inflammatory and pro-tumorigenic because of the increased circulation of cytokines and growth factors, promoting tumorigenesis and tumor progression [24], which might be the underlying reason of our results. The mechanisms by which visceral obesity is thought to promote tumorigenesis are manifold, including alterations in adipokine secretion, as well as hyperinsulinemia and subsequent stimulation of insulin-like growth factor-1 axis [25]. Besides, the abundant inflammatory cells in visceral adiposity create systemic inflammation and a pro-tumorigenic environment [25].

The widely accepted and used BMI criteria is not suitable for determining visceral obesity, as within each category of BMI there could be substantial individual variations in visceral adiposity (clear shown in Fig. 2) [13]. Additionally, Asian adults generally have a slighter physique than Western population, with a less central body weight distribution [26], so VAT might be a more accurate measure of obesity in Asian individuals than BMI. In our series, BMI correlated well with total adipose tissue, but not with VAT%. Therefore, we suggested that VAT% might be an important surrogate for obesity in addition to BMI. In the current time, CT image-based quantitative measurement of body adiposity is still primarily reserved for research purpose. With the introduction of commercially available software that does not require special training, VAT assessment using CT scan could be simple and accurate [27]. However, what is unknown is the acceptable threshold level of VAT, above which it begins to detrimentally affect metabolic and inflammatory processes, ultimately inducing tumor progression [28]. We dichotomized the study population according to the median VAT% value. The lack of appropriate threshold might hinder the ability to make useful correlation between visceral obesity and markers of tumor phenotype.

Our study has limitations inherent to its retrospective design, including non-randomization, possible selection bias and completeness of previously recorded data. In addition, this cohort was restricted to a single institution, one of the leading cancer centers in China. Thus, there is a possibility of patient selection bias, which might hinder the extrapolation of our results to other population. A large multicenter study would help to confirm and validate our findings.

## Conclusions

Visceral obesity defined by VAT% proved to an independent factor for lymph node metastasis in endometrial cancer, along with the grade 3 disease and presence of lympho-vascular invasion. Patients with high VAT% tended to be old and have extrauterine disease. BMI was associated with total adiposity but not with VAT%.

## Availability of data and materials

The data involved in the current study are available upon request. Anyone who is interested in the information should contact docwuxh@hotmail.com.

## Abbreviations

BMI: body mass index
CI: confidence interval
CT: computerized tomography
FIGO: International Federation of Gynecology and Obstetrics
HR: hazard ratio
SAT: subcutaneous adipose tissue
VAT: visceral adipose tissue
VAT%: percentage of visceral adipose tissue
